# A Young Man With Acute Kidney Injury and Uveitis: An Unusual Presentation and Case Report

**DOI:** 10.7759/cureus.25927

**Published:** 2022-06-14

**Authors:** Junaid Iqbal, Mohammed T Alassafi, Faisal M Alashaikh, Mohammed M Alhafi, Abdulrahman N Abohaimid

**Affiliations:** 1 Department of Medicine/Division of Nephrology, King Abdulaziz Medical City, Ministry of National Guard Health Affairs, Riyadh, SAU; 2 Department of Medicine, King Saud Bin Abdulaziz University for Health Sciences, Riyadh, SAU

**Keywords:** tinu, acute kidney disease, acute kidney injury, tubulointerstitial nephritis and uveitis, tubulointerstitial nephritis with uveitis

## Abstract

Tubulointerstitial nephritis with uveitis (TINU) is an uncommon autoimmune syndrome that involves multiple systems. It usually presents with acute kidney injury (AKI) and unilateral or bilateral uveitis. We present a 15-year-old male, fit and well, who attended emergency room (ER) with a four-month history of epigastric pain associated with nausea, vomiting, and weight loss of 10 kg. Initial clinical and laboratory evaluation confirmed AKI. A diagnostic kidney biopsy confirmed acute tubulointerstitial nephritis, and a slit-lamp examination confirmed acute left anterior uveitis consistent with a diagnosis of TINU. He was initially treated with corticosteroids which resulted in prompt resolution of AKI; however, uveitis persisted necessitating the addition of further immunosuppressants.

## Introduction

Tubulointerstitial nephritis and uveitis (TINU) syndrome is an uncommon disease that affects the renal tubular cells and eyes, resulting in tubulointerstitial nephritis and unilateral or bilateral uveitis. This syndrome was first described by Dobrin et al. in 1975 [1]. The underlying pathophysiology remains unclear to date; however, TINU is believed to be triggered by infection or with the use of certain medications (antibiotics or nonsteroidal anti-inflammatory drugs) [2]. This syndrome is reported to have a female predominance (65% of cases) and a median age of 17 [3]. It is reported that TINU is responsible for 5% of the cases of acute tubulointerstitial nephritis [4]. However, only less than 1% of uveitis cases had been related to TINU syndrome [5]. It is vital to be aware of this syndrome to ensure early diagnosis, prompt treatment, and better renal and patient outcomes.

## Case presentation

A 15-year-old boy was referred to the emergency room (ER) by a primary healthcare clinic due to an incidental finding of elevated serum creatinine. He attended the primary healthcare clinic recently for intermittent vomiting and abdominal pain. In ER, the patient described a four-month history of off and on epigastric pain associated with nausea and vomiting. Vomiting was intermittent, up to once a day, and associated with malaise and reduced appetite. He reported up to 10 kg of weight loss during the same period. Left eye redness, with no pain or discharge, was noticed five days prior to ER presentation. The patient denied flank pain, dysuria, visible hematuria, oliguria, rectal bleeding, diarrhea or constipation, joint pains, mouth ulcers, skin rash, fever, lower limb or periorbital edema, chest pain, or any recent upper respiratory tract symptoms.

On examination, the patient was alert and conscious. He was afebrile and had a blood pressure of 137/77 mmHg while sitting, checked with an appropriate size cuff on his right arm. The heart rate was 100 beats per minute, the respiratory rate was 20 breaths per minute, and the temperature was 36.9 degrees Celsius. He was euvolemic. Minimal tenderness was noted in the epigastric and left upper quadrant areas. His left eye was congested but with no discharge. There was also a decreased visual acuity of the left eye (20/28) with normal intraocular pressure. Left anterior nongranulomatous uveitis was later diagnosed.

Laboratory workup revealed a serum creatinine level of 303 µmol/L, potassium 3.4 mmol/L, sodium 133 mmol/L, and mild anemia (hemoglobin (Hb) of 106 g/L). Lactate dehydrogenase and C-reactive protein (CRP) were elevated to 377 U/L and 10 mg/L, respectively, while erythrocyte sedimentation rate (ESR) was normal. Urinalysis showed a pH level (6.0), urine specific gravity (1.011), proteinuria (50 mg/dL), and a spot urine protein/creatinine ratio (0.75 g/g).

Renal ultrasound demonstrated both kidneys of normal size and echogenicity without stone or hydronephrosis. An ultrasound renal Doppler did not show any renovascular disease. An unenhanced computerized tomography (CT) of the abdomen and pelvis was unremarkable. Further clinical and laboratory evaluation did not reveal any evidence of underlying bacterial, parasitic, or viral infection. Similarly, spondyloarthropathy or other rheumatological diseases associated with uveitis such as ankylosing spondylitis, psoriatic arthritis, and reactive arthritis were absent.

**Figure 1:**
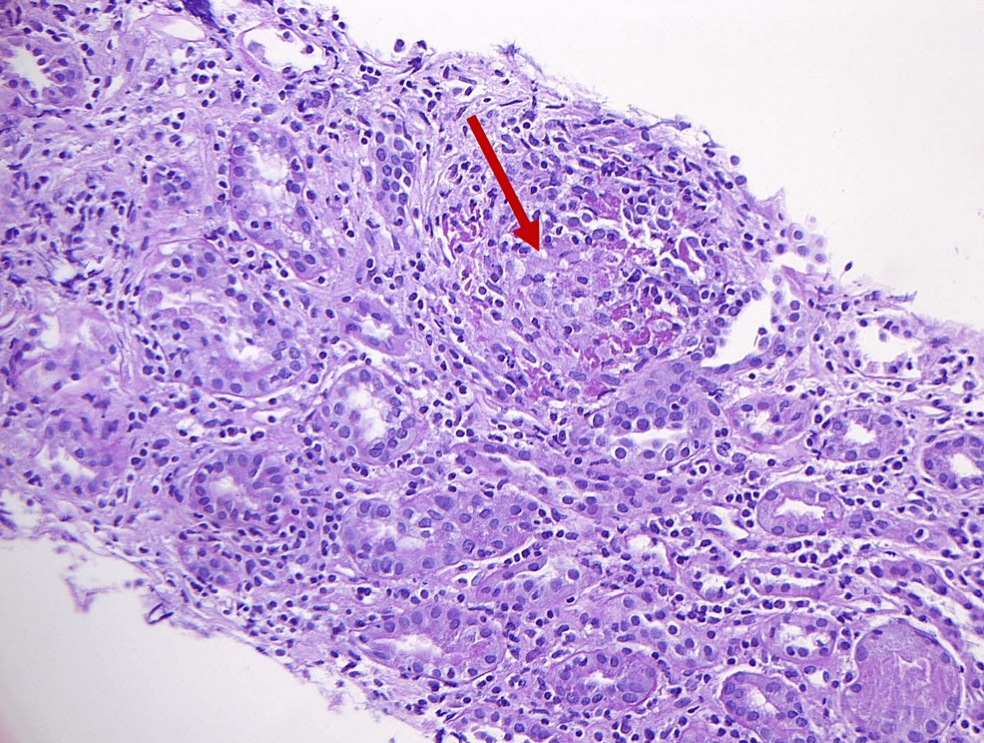
Renal biopsy shows interstitial inflammation with ill-defined granuloma (red arrow) around ruptured tubule (Periodic acid-Schiff stain, X20)

**Figure 2:**
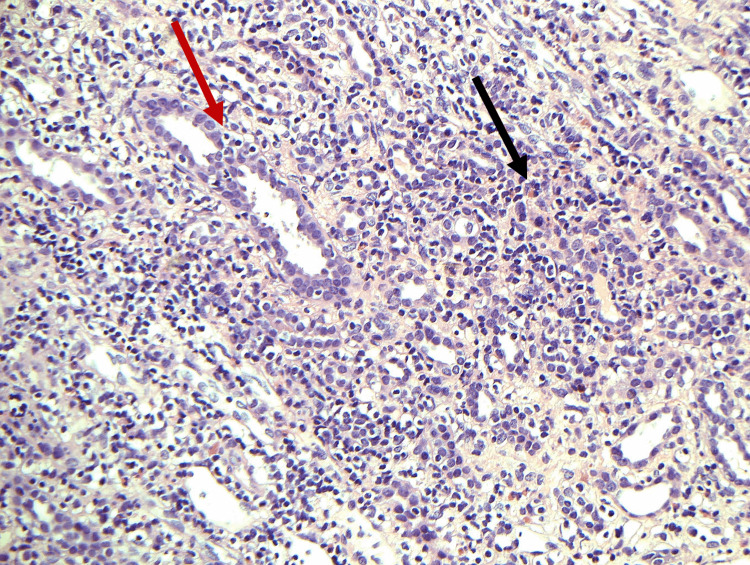
The interstitial infiltrate is tense consisting mostly of lymphocytes attacking tubules (red arrow), plasma cells, and scattered eosinophil (black arrow) (hematoxylin and eosin, X20)

The patient was admitted under the care of Nephrology and was counseled regarding acute kidney injury (AKI), presence of proteinuria, and a diagnosis of uveitis. An ultrasound-guided percutaneous left kidney diagnostic biopsy was performed which confirmed tubulointerstitial nephritis with granulomatous inflammation and foreign body giant cell reaction (Figures 1, 2). Immunofluorescence and IgG4 staining were negative.

The patient was commenced on oral prednisolone 60 mg once a day promptly. Prednisolone was gradually tapered over the following two months to 25 mg once a day. Due to a lack of significant improvement in uveitis, mycophenolate mofetil (MMF) 500 mg twice daily, prednisolone eye drops, and mydriatics were added by Ophthalmology. Uveitis failed to settle promptly requiring an increase in the dose of prednisolone to 50 mg once a day in a subsequent review a month later. The importance of adherence to prescribed medications was reiterated to the patient and discussed with parents.

At a six-month review, the patient’s serum creatinine had returned to 95 µmol/L, and he was continued on prednisolone 10 mg once a day and MMF 1,500 mg daily in divided doses. Due to active and persistent uveitis despite steroids and immunosuppressive medication, infliximab was added, following a discussion with Ophthalmology and Rheumatology. At his most recent visit, the patient’s uveitis has resolved, and his serum creatinine remains stable at 96 µmol/L with the resolution of proteinuria to 0.15 g/g (urine albumin: creatinine ratio 2 mg/g). He continues his follow-up in Nephrology, Ophthalmology, and Rheumatology outpatients.

## Discussion

The syndrome of tubulointerstitial nephritis and uveitis is an uncommon autoimmune disease that involves multiple systems. It may be triggered in response to microbial pathogens, drugs, and involvement of human leukocyte antigen (HLA)-related genetic predisposition [6]. We have reviewed four previously published articles and case reports (Table 1). The age of diagnosis varied from 14 to 49 years of age. Three out of four patients were female [4,6,7]. The clinical presentation varied among the cases ranging from reduced visual acuity and/or burning sensation in the eyes with a blurred vision to lumbar pain, transitory dark urine, anorexia, weight loss, significant hypertension, progressive renal dysfunction, and periorbital and leg edema [4,6-8]. Our patient presented with left eye congestion with no pain or discharge and weight loss.

**Table 1:** Review of previously published case reports and articles Hb: hemoglobin, TINU: tubulointerstitial nephritis with uveitis.

Author	Age	Gender	Clinical presentation	Ophthalmology	Laboratory	Urinalysis	Method of diagnosis	Treatment	Follow-up and prognosis
Lopes et al. [4]	49 years old	Female	Lumbar pain, transitory dark urine, asthenia, anorexia, weight loss, and leg and periorbital edema	Unilateral anterior uveitis	Elevated serum creatinine (350.06 µmol/L), normocytic normochromic anemia (Hb of 101 g/L)	Hematuria and proteinuria (1,044 mg/24 h) without cellular casts	Kidney biopsy showed diffuse mononuclear cell interstitial infiltrates that are consistent with acute tubulointerstitial nephritis	Oral prednisone	Clinical improvement at six months with renal function recovery. However, after dose tapering at eight months, the patient presented with unilateral anterior nongranulomatous uveitis
Clive and Vanguri [6]	30 years old	Female	Burning sensation in the eyes associated with blurred vision	Bilateral acute uveitis	Elevated serum creatinine (159.12 µmol/L)	Unremarkable	Percutaneous kidney biopsy showed tubulointerstitial nephritis with predominantly lymphocytes	Prednisone, lisinopril, topical ophthalmic prednisone	One year after treatment, the uveitis resolved, and serum creatinine dropped to 79.56-88.4 µmol/L
Petek et al. [7]	14 years old	Female	One week of eye redness, pain, epiphora, and loss of visual acuity	Unilateral anterior uveitis	Elevated serum creatinine (80 µmol/L), mild normocytic anemia (Hb of 113 g/L)	Mild proteinuria (0.38 g/day), microalbuminuria (urine albumin-to-creatinine ratio of 58 mg/g), elevated α1-microglobulin (urine α1-microglobulin-to-creatinine ratio of 3.24 mg/g), glycosuria (1+)	Histopathology showed focal tubulointerstitial nephritis	Topical cycloplegic on both sides. Methylprednisolone, pantoprazole, trimethoprim-sulfamethoxazole, topical ocular therapy (scopolamine, dexamethasone, nepafenac), ramipril	At a three-month follow-up, the patient’s ocular symptoms improved. At two and a half years of follow-up, the patient denied any further exacerbation of ocular symptoms and her blood pressure was normal.
Zhao et al. [8]	37 years old	Male	Significant hypertension and progressive renal dysfunction	Fundal hemorrhage and malignant hypertension. New-onset bilateral uveitis after treatment cessation by four months	Elevated serum creatinine (935.9 µmol/L) rapidly increased to 2,640 µmol/L in two weeks with urine volume less than 300 mL/day	Mild proteinuria (1.31 g/24 h). Urine/blood osmolarity of 217/307. Renal glycosuria (urine/glucose: 3+/5.32 mmol/L). Elevated α1-microglobulin level (214 mg/L)	Kidney biopsy and electron microscopy confirmed the diagnosis of thrombotic microangiopathy superimposed by drug-induced acute tubulointerstitial nephritis	Hemodialysis, telmisartan, oral prednisone	After treatment for four months, serum creatinine levels gradually decreased. At a 10-month follow-up (four months after prednisone cessation), the patient developed acute kidney disease (TINU recurrence)
Iqbal et al (Our case)	15 years old	Male	Left eye redness with no pain or discharge. Weight loss of about 10 kg	Left anterior nongranulomatous uveitis	Elevated serum creatinine (303 µmol/L). Elevated lactate dehydrogenase (377 U/L). Anemia (Hb of 106 g/L)	Proteinuria (50 mg/dL)/urine protein/creatinine ratio of 0.75 g/g	Percutaneous left kidney biopsy showed tubulointerstitial nephritis with granulomatous inflammation and foreign body giant cell reaction	Oral prednisolone, topical ophthalmic prednisolone, mydriatics, mycophenolate mofetil, infliximab	At a six-month follow-up, the lab findings were within normal levels, but uveitis was still active. So, infliximab medication was initiated. On the last visit, the patient was documented as inactive tubulointerstitial nephritis with uveitis

Certain precipitating factors for TINU are reported in three of the cases [6-8]: firstly, upper respiratory tract infection associated with mild conjunctivitis and found prior to the diagnosis of TINU [6]; secondly, the use of herbal medications in the preceding two weeks prior to a finding of elevated serum creatinine level and the diagnosis of TINU [8]; and thirdly, the presence of multiple chronic diseases including hypertension, dyslipidemia, obesity, and insulin resistance with a sedentary lifestyle [7]. Ophthalmic findings were present in all cases. Two of which were unilateral uveitis [4,8]. Similarly, our case had left anterior uveitis. The other two were bilateral anterior uveitis [6,8]. In one of the aforementioned case reports, new-onset bilateral uveitis occurred in a patient after prednisone was tapered to discontinuation at four months [8]. Treatments used to treat uveitis included topical ophthalmic prednisone, topical cycloplegic, and topical ocular therapy, such as scopolamine, nepafenac, and dexamethasone.

Laboratory findings included elevated serum creatinine levels in all the cases including ours. One of the reported cases had a severely elevated creatinine level of 2,640 µmol/L with a urine volume of less than 300 mL/day which necessitated the initiation of acute hemodialysis [8]. In addition, mild anemia was found in two of the cases like ours [4,7]. Urinalysis showed proteinuria in three cases like our case [4,7,8], one of whom had hematuria without cellular casts [4]. Two cases revealed elevated α1-microglobulin and glucosuria [7,8]. Our patient’s urinalysis showed proteinuria (50 mg/dL) and spot urine protein/creatinine ratio (0.75 g/g). However, the urinalysis in one case was unremarkable [6].

The diagnosis was confirmed with a renal histopathological examination in all the cases. Immunofluorescence was negative in two of the cases same as our case [7,8], but not available in the other two [4,6]. Ophthalmic finding of either unilateral or bilateral uveitis along with the presence of AKI, proteinuria, and elevated blood pressure should raise suspicion of tubulointerstitial nephritis and uveitis [4,6-8]. Oral prednisolone was used in three cases, with a slow taper, once renal function started to improve [4,6,8], Angiotensin-converting enzyme inhibitors (ACEi) and angiotensin receptor blockers (ARBs) were used to control [6-8]. Topical ocular therapy was administered in addition to systemic steroids, as mentioned earlier. Only one case necessitated the need for hemodialysis due to a severe reduction in urine volume of 300 mL/day and the development of AKI stage 3 [8]. Our patient was treated with oral prednisolone, mycophenolate mofetil, ophthalmic topical prednisolone, and mydriatics. Infliximab was initiated, following a clinical review in rheumatology outpatients, due to persistent uveitis at six months.

Advanced disease at the time of presentation and diagnosis and the late initiation of pharmacological management are two primary factors responsible for variations in clinical manifestation and the overall outcome for patients with TINU. The prognosis of TINU is usually favorable, and patients typically respond to pharmacological management within a few months of treatment initiation [4,6-8]. However, there is a risk of disease recurrence once steroids are being tapered or weaned off [8]. This may warrant a course of oral corticosteroids for a longer duration. [8]. Our patient, at a six-month follow-up, was on oral prednisolone and mycophenolate mofetil. His renal functions had returned to his baseline; however, uveitis remained active and persistent despite the usage of corticosteroids and immunosuppressants. This required the addition of infliximab, following discussion with Rheumatology, which resulted in improvement in eye disease. The patient’s uveitis was quiescent on his most recent outpatient follow-up visit.

## Conclusions

Tubulointerstitial nephritis and uveitis is an uncommonly seen disease entity. A new elevation in serum creatinine combined with the presence of acute uveitis should alert the clinician to the possibility of TINU, especially in young individuals. First-line treatment involves topical and oral corticosteroids. The renal outcomes in TINU are usually excellent, but eye symptoms and uveitis can take time to improve and are fraught with the risk of recurrence.
